# 

**Published:** 1882-10

**Authors:** 


					﻿/OCTOBER, 1882.
TWENTY-NINTH YEAR.
HALL’S
JOURNAL
OF
HEALTH
E. H. GIBBS, A.M.,	Editor,
No. 135 EIGHTH STREET (Near Broadway), NEW YORK
TERMS: $2 a Year, or $1 for Six Months. Single number, 20 ctsJ
m sUflAnd.olfiM Matter In the Poftt Offiee in New York.
				

